# Xenogeneic Graft-versus-Host-Disease in NOD-*scid* IL-2Rγ^null^ Mice Display a T-Effector Memory Phenotype

**DOI:** 10.1371/journal.pone.0044219

**Published:** 2012-08-28

**Authors:** Niwa Ali, Barry Flutter, Robert Sanchez Rodriguez, Ehsan Sharif-Paghaleh, Linda D. Barber, Giovanna Lombardi, Frank O. Nestle

**Affiliations:** 1 St. John's Institute of Dermatology, King's College London and NIHR Biomedical Research Centre, London, United Kingdom; 2 MRC Centre for Transplantation, King's College London and NIHR Biomedical Research Centre, London, United Kingdom; 3 Department of Haematological Medicine, King's College London, London, United Kingdom; University of California, San Francisco, United States of America

## Abstract

The occurrence of Graft-versus-Host Disease (GvHD) is a prevalent and potentially lethal complication that develops following hematopoietic stem cell transplantation. Humanized mouse models of xenogeneic-GvHD based upon immunodeficient strains injected with human peripheral blood mononuclear cells (PBMC; “Hu-PBMC mice”) are important tools to study human immune function *in vivo*. The recent introduction of targeted deletions at the interleukin-2 common gamma chain (IL-2Rγ^null^), notably the NOD-*scid* IL-2Rγ^null^ (NSG) and BALB/c-*Rag2* ^null^ IL-2Rγ^null^ (BRG) mice, has led to improved human cell engraftment. Despite their widespread use, a comprehensive characterisation of engraftment and GvHD development in the Hu-PBMC NSG and BRG models has never been performed in parallel. We compared engrafted human lymphocyte populations in the peripheral blood, spleens, lymph nodes and bone marrow of these mice. Kinetics of engraftment differed between the two strains, in particular a significantly faster expansion of the human CD45^+^ compartment and higher engraftment levels of CD3^+^ T-cells were observed in NSG mice, which may explain the faster rate of GvHD development in this model. The pathogenesis of human GvHD involves anti-host effector cell reactivity and cutaneous tissue infiltration. Despite this, the presence of T-cell subsets and tissue homing markers has only recently been characterised in the peripheral blood of patients and has never been properly defined in Hu-PBMC models of GvHD. Engrafted human cells in NSG mice shows a prevalence of tissue homing cells with a T-effector memory (T_EM_) phenotype and high levels of cutaneous lymphocyte antigen (CLA) expression. Characterization of Hu-PBMC mice provides a strong preclinical platform for the application of novel immunotherapies targeting T_EM_-cell driven GvHD.

## Introduction

Allogeneic hematopoietic stem cell transplantation (HSCT) can be a potential curative therapy for a variety of malignant and non-malignant hematological diseases. While beneficial graft-versus-tumour immune responses are elicited by transferred donor derived T-cells, the alloreactivity of these cells against HLA disparities and minor antigens can result in the initiation of graft-versus-host disease (GvHD). The development of GvHD is a potentially life threatening complication that often commences with an acute phase–with surviving patients then being affected by the chronic form of the disease [1]. Lymphocyte depletion is used to reduce the GvHD alloresponse [2], [3] but this non-specific approach leaves patients at considerable risk of complications such as infection or cancer relapse. Therefore, better intervention therapies to prevent GvHD are required.

The generation of immunodeficient mice bearing a targeted mutation in the IL-2 receptor common gamma chain (IL-2Rγ^null^), notably the NOD-*scid* IL-2Rγ^null^ (abbreviated NSG) and BALB/c-*Rag2* ^null^ IL-2Rγ^null^ (abbreviated BRG) that lack T- B- and NK- cells, permits acceptance of human cells and tissues [4], [5]. These strains engraft readily following intravenous injection of human peripheral blood mononuclear cells (PBMCs; abbreviated as Hu-PBMC mice). Hu-PBMC NSG and Hu-PBMC BRG mice are currently used as reliable models of human-into-mouse xenogeneic GvHD where the engrafted cells are amenable to regulation by therapeutic agents or adoptively transferred human immune cells such as regulatory T-cells (Tregs) or tolerogenic dendritic cells [6]–[12]. These mice are therefore useful models for pre-clinical evaluation of novel GvHD therapies. GvHD development in these models is dependent upon human PBMC xeno-reactivity with foreign host major histocompatibility (MHC) class I and class II [11], resembling HLA mismatched HSCT in clinical GvHD where donor cell alloreactivity is initiated by recognition of foreign MHC antigens expressed by recipient cells [13]. In human acute GvHD, the characteristic involvement of specific organs strongly suggests that immune cell trafficking is essential to the pathophysiology of disease. This has been linked to the expression of homing receptors that direct T-cells to specific extralymphoid tissues. Recent reports have pointed towards alterations of T-cell expression of the specific skin homing marker cutaneous lymphocyte antigen (CLA) as a predictive immunobiological marker of acute GvHD in the periphery of HSCT patients [14], [15]. Such human studies employ analysis of peripheral blood because patient tissues are usually inaccessible due to obvious ethical and technical constraints. The opportunity to examine via flow cytometry the T-cell composition and reconstitution of lymphoid organs in GvHD patients is crucial for developing preventative strategies.

In this work, we describe the first systematic comparative characterization of tissue lymphocyte populations and kinetics between Hu-PBMC NSG and BRG mice, the two most widely used immunodeficient strains to study xenogeneic GvHD. The major aim of our study was to firstly determine the differential rate of GvHD development, which is a critical factor to consider when selecting a model to assess the efficacy of novel immunotherapies rapidly. These data show that NSG mice are more optimal and robust models of GvHD with a significantly faster rate of disease development. We then chose Hu-PBMC NSG mice to perform extensive tissue phenotyping of naive and memory T-cell subsets and CLA expression, which has not been properly defined in these humanized mice. The characterization of engrafted cells in these Hu-PBMC models can be used as pre-clinical platforms to develop novel therapeutic strategies for clinical application.

## Materials and Methods

### Ethics Statement

This study involving the use of human participants was specifically approved by the institutional review board of Guy's Hospital (Guy's Research Ethics Committee, Ethics Committee Code: 06/Q0704/18) and conducted in accordance with the Helsinki Declaration. Informed written consent was obtained from all healthy controls prior to enrolment into the study. Animals were maintained under specific pathogen-free conditions and all animal experiments were specifically approved by the Institutional Committees on Animal Welfare of the United Kingdom Home Office (the Home Office Animals Scientific Procedures Act, 1986).

### Immunodeficient Mice

Male and female NOD/scid/*IL-2Rγ^−/−^* mice (NOD.cg-Prkdc^scid^Il2rg^tm1Wjl^/SzJ abbreviated to NSG; obtained from The Jackson Laboratory) and BALB/c RAG2^−/−^γ_c_^−/−^ mice (abbreviated to BRG; kindly provided by Prof. Adrian Hayday, The London Research Institute, Cancer Research UK, London, England, UK; and P. Gorer, Department of Immunobiology, King's College School of Medicine at Guy's Hospital, London, England, UK) were used between 6–12 weeks of age.

### Histological analysis of mouse skin

Mouse skin was harvested and snap frozen in optimum cutting temperature (OCT) solution over pre-cooled isopentane. Frozen blocks were then cut at 6–8 µm and stored at −80°C until use. Sections were air dried, fixed in cold acetone. Non-specific Fc receptor binding was measured using isotype non-binding control antibodies. Immunohistochemical staining was performed using an avidin-biotin methodology with mouse monoclonal antibodies specific for human CD45 (clone HI30; eBioscience). Dilutions were conducted as per manufacturer's guidance. An appropriate biotinylated secondary antibody was then added at a 1∶200 dilution and left for 60 min at room temperature. Sections were treated with the ABC Vectastain Elite kit (VectorLabs, Peterborough UK) and the colour developed using 3-diaminobenzidine then counterstained with hematoxylin for visualisation by light microscopy.

### Humanised mouse model of xeno-Graft-versus-Host Disease (GvHD)

PBMCs were isolated from buffy coats (provided by the National Blood Transfusion Centre; South Thames, Tooting, UK), by density gradient centrifugation over Lymphocyte Separation Medium (PAA), and then resuspended in 200 µl of PBS in insulin syringes (VWR). Xenogeneic GvHD was induced by intravenous injection of 10^7^ human PBMCs via the tail vein into unconditioned adult NSG and BRG mice. In the lightly irradiated xeno-GvHD model, NSG mice were first irradiated with 2.4 Gy and injected 24 hours later with human PBMCs. In all experiments, fresh human PBMC were used and were never freeze-thawed. Animals that developed clinical symptoms of GvHD (>15% weight loss, hunched posture, fur loss, reduced mobility, tachypnea) were sacrificed and an end point of survival was recorded for all Hu-PBMC mice.

### Antibodies and flow cytometry

The following antibodies were used in dilutions according to manufacturers instructions: FITC conjugated anti-human CD62L (Invitrogen), PE conjugated anti-human CD27 (eBioscience), PE conjugated anti-human CD25 (BD), PE conjugated anti-human CLA (Miltenyi), PE-Cy5.5 conjugated anti-human CD3 (Invitrogen), PE-Cy5.5 conjugated anti-human CD20 (Invitrogen), PE-Cy7 conjugated anti-mouse CD45 (eBioscience), PE-TR conjugated anti-human CD4 (Invitrogen), APC conjugated anti-human CD8 (Invitrogen), APC-Cy7 conjugated anti-human CD45 (eBioscience), and Pacific Blue conjugated anti-human CD45RO (BD). Single-cell suspensions from spleens, bone marrows and lymph nodes were prepared in PBS containing 1% fetal bovine serum (Sigma) and 2 mM EDTA (Invitrogen). Blood was collected via tail vein bleeding using EDTA-coated capillary tubes. CountBright absolute counting beads (Invitrogen) were added to spleen samples prior to acquisition and absolute numbers of cells calculated accordingly.

### Lymph nodes in NSG and BRG mice are highly atrophic, however during xeno-GvHD progression distinct lymph node structures become apparent in the axial and brachial positions that were harvested and pooled for cellular analysis

Single cell suspensions were incubated in anti-mouse CD16/32 (clone 2.4G2; BD) for 5 min at 4°C to block nonspecific Fc binding and antibodies were then added in the appropriate combinations. Labeled cells were washed, and at least 100,000 events were acquired were acquired with a BD FACS Canto (BD) and data were analyzed by FlowJo (TreeStar) software. For all analyses, anti-mouse CD45 staining was performed to exclude murine host cells from analysis, and only those cells that were human CD45 positive and mouse CD45 negative were included in the final calculation of reported values [16]. CD3^+^ CD4^+^ and CD3^+^ CD8^+^ T-cell subsets were defined as CD45RO^−^CD27^+^ naïve, CD45RO^+^ CD27^+^ CD62L^+^ central memory, CD45RO^+^ CD27^+^ CD62L^−^ effector memory, CD45RO^+^ CD27^−^ effectors and CD45RO^−^ CD27^−^ terminal effectors (please see Supplementary Figure 1 for representative gating strategies).

**Figure 1:**
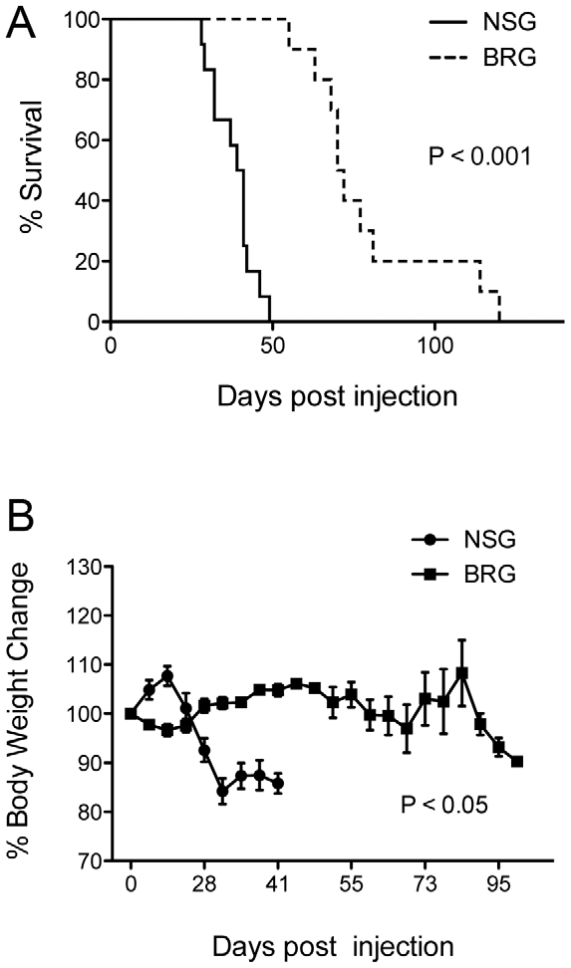
NSG mice develop GvHD at a faster rate than BRG mice. 10^7^ freshly isolated human PBMCs were injected intravenously via the tail vein into adult 6–10 week old NSG and BRG immunodeficient mice. (A) Survival of NSG mice [median survival time (MST) = 40 days, *n* = 12, 4 PBMC donors] was significantly shorter than that of BRG mice (MST = 71, *n* = 10, 3 PBMC donors). (B) The average weight is shown as a percentage of starting weight. (A) Log-rank Mantel-Cox Test and (B) unpaired t test. Each data point represents the mean ±SEM.

### Statistical analysis

Statistical analysis was performed using GraphPad Prism version 4.0 (GraphPad Software). All measures of variance are presented as standard error of the mean (SEM). Results were assessed for normal Gaussian distribution and then analyzed by Mann-Whitney non-parametric t test, one-way ANOVA test, two-way ANOVA, or Mantel-Cox as indicated. Data were considered to be significantly different when P<0.05, P<0.01, or P<0.001 represented in the figures and tables as *, **, or ***, respectively.

## Results

### Engraftment kinetics of human cells in Hu-PBMC NSG and BRG mice

**Figure 2:**
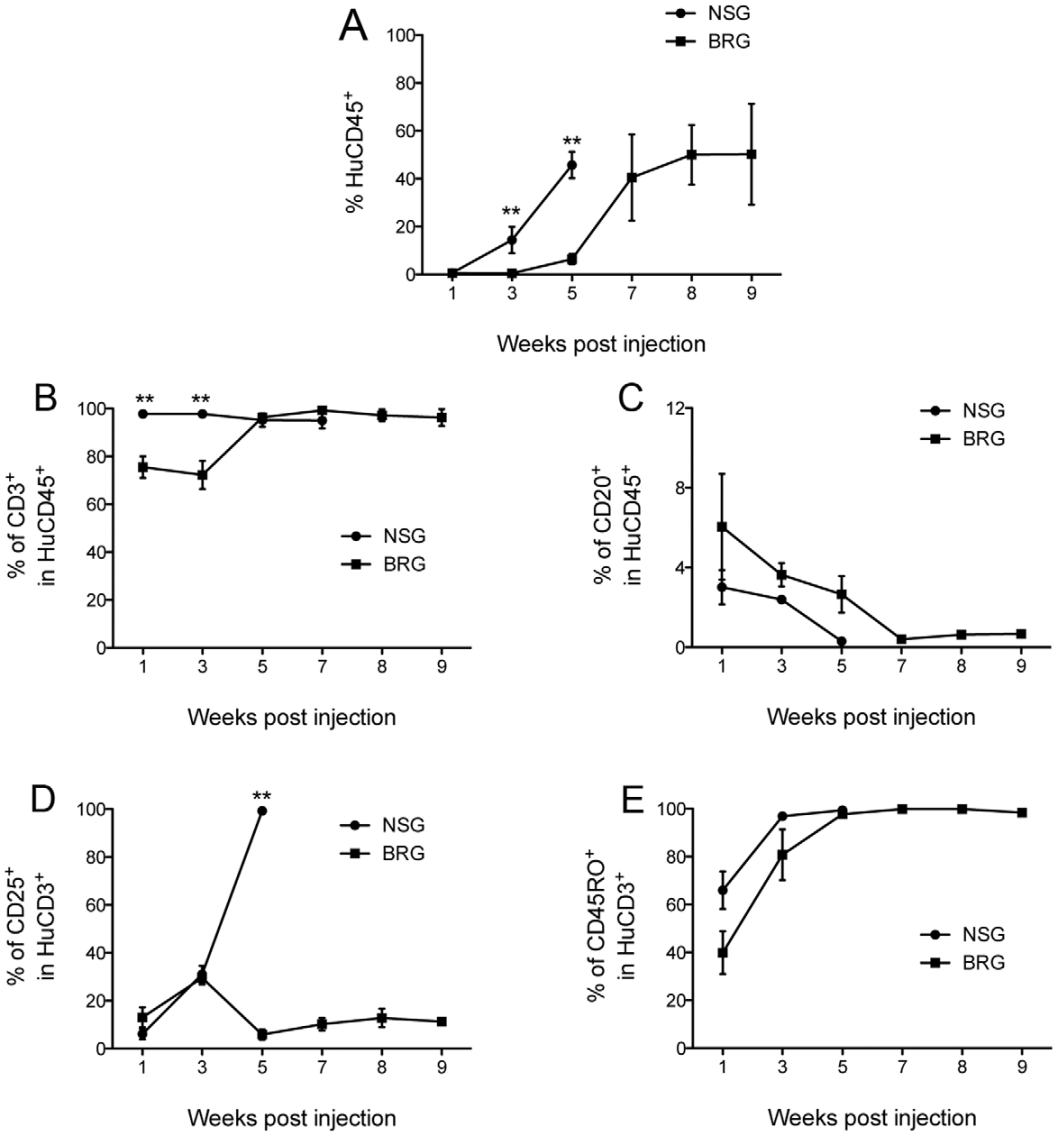
Kinetics of human cell engraftment in NSG and BRG mice. Peripheral blood was collected from Hu-PBMC mice bi-weekly and stained with anti-human specific antibodies for human (A) CD45, (B) CD3, (C) CD20, (D) CD25, and (E) CD45RO and quantified by flow cytometry. 2-way ANOVA test was performed. Each data point represents the average (*n* = 4 NSG and BRG mice respectively, 2 PBMC donors) ±SEM values.

Several reports have shown that NSG and BRG mice engraft efficiently with human PBMC in the absence of host pre-conditioning [6], [11], [17]–[19], but a systematic comparison between the two strains has never been performed. To this end, NSG and BRG mice at 6–12 weeks of age were injected intravenously with 10^7^ human PBMC via the tail vein. Both strains developed GvHD consistently with accelerated weight loss and significantly faster disease development in NSG mice [median survival time (MST) = 40 days vs. 71 days in Hu-PBMC BRG mice; P<0.001; Fig. 1A–B]. Kinetics of human cell engraftment in the peripheral blood was evaluated bi-weekly until mice developed GvHD. At which point, spleens, lymph nodes, and bone marrow were also harvested for analysis. Levels of human CD45^+^ cells in the periphery of NSG mice at three and five weeks post injection were significantly higher than those achieved in BRG mice (P<0.01), where expansion was much slower (Fig. 2A). In agreement with previous studies [18], the majority of the engrafted cells in NSG mice were CD3^+^ (>95%; Fig. 2B), whereas in BRG mice these proportions were significantly lower (<80%) at early time points (P<0.01; Fig. 2B).

**Figure 3:**
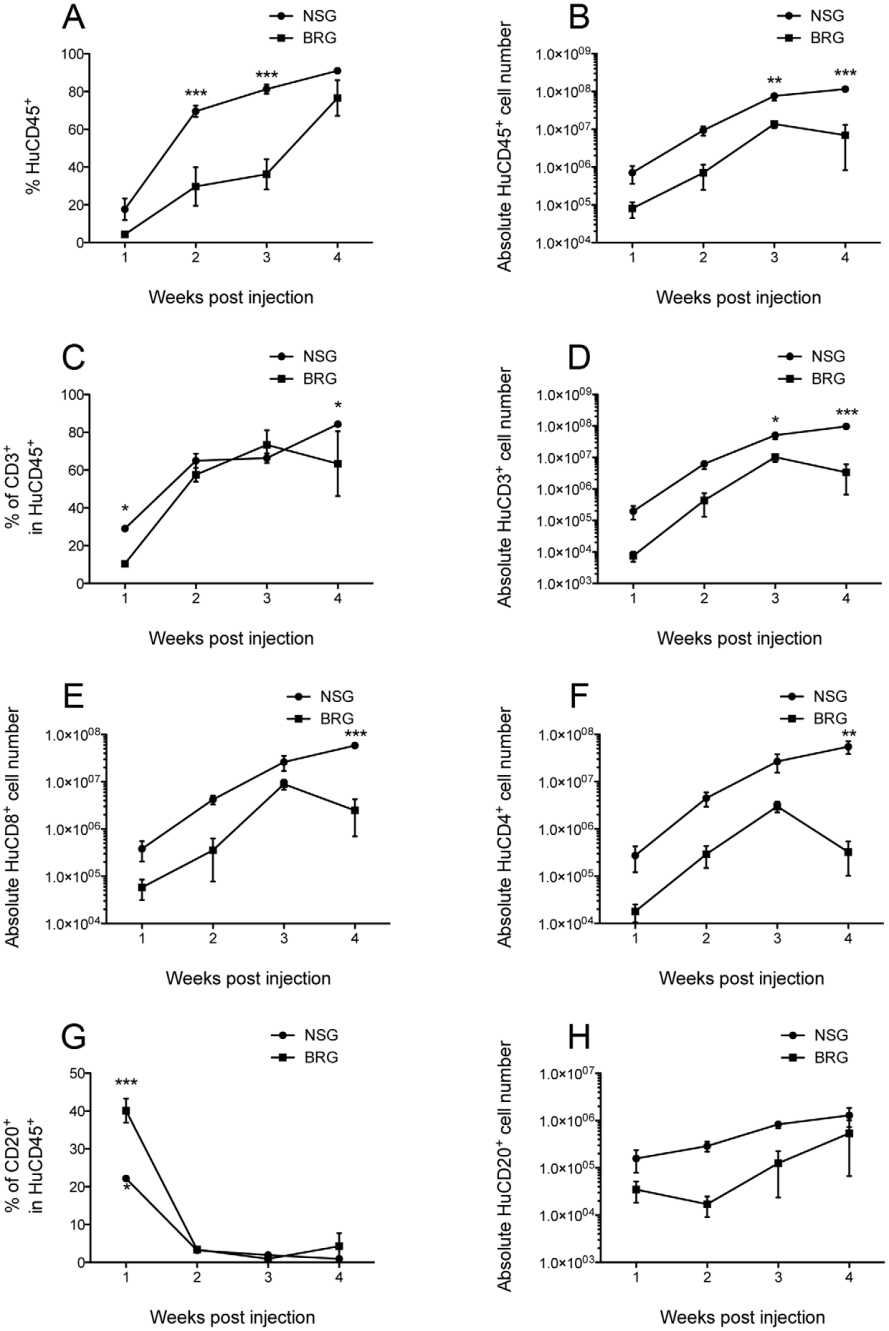
Kinetics of splenic lymphocyte engraftment in Hu-PBMC NSG and BRG mice. Cells were harvested from the spleens of Hu-PBMC NSG and BRG mice at weekly intervals following transfer and stained with anti-human specific antibodies for mouse CD45 and for human CD45, CD3, CD4, and CD20. Samples were analysed by flow cytometry to calculate the % of human (A) CD45^+^, (C) CD3^+^ and (G) CD20^+^, the absolute accumulation of human (B) CD45^+^, (D) CD3^+^, (E) CD8^+^, (F) CD4^+^ and (H) CD20^+^ cells. 2-way ANOVA test was performed. Data are compiled from 2 independent experiments (NSG n = 4–5 per time point and BRG n = 4 day 7–21 or n = 2 day 28, 2 PBMC donors).

To confirm the difference in human cell expansion in the periphery of the two strains, we then performed kinetic analysis of chimaerism in the spleen during the first four weeks of human PBMC engraftment. Indeed, very much mirroring peripheral blood reconstitution in these mice, there was a significantly higher proportion of human CD45^+^ cells in NSG mice as compared to BRG mice at all four weekly time points measured post PBMC transfer (Fig. 3A). The overall cellularity of the spleens was also greatly increased (Fig. 3B) mainly as a result of a dramatic accumulation of donor T-cells (Fig. 3C and D). CD4 and CD8 T-cell subsets accumulated in both NSG and BRG mice but with significantly greater accumulation in NSG mice (Fig. 3E and F). The proportion of CD20^+^ B-cells contracted with time, reflecting the huge expansion in T-cells, (Fig 3G) however, the absolute number of B-cells increased during the 4-week time course. Once again the absolute splenic accumulation of B-cells was greater in NSG mice than in BRG recipients.

**Figure 4:**
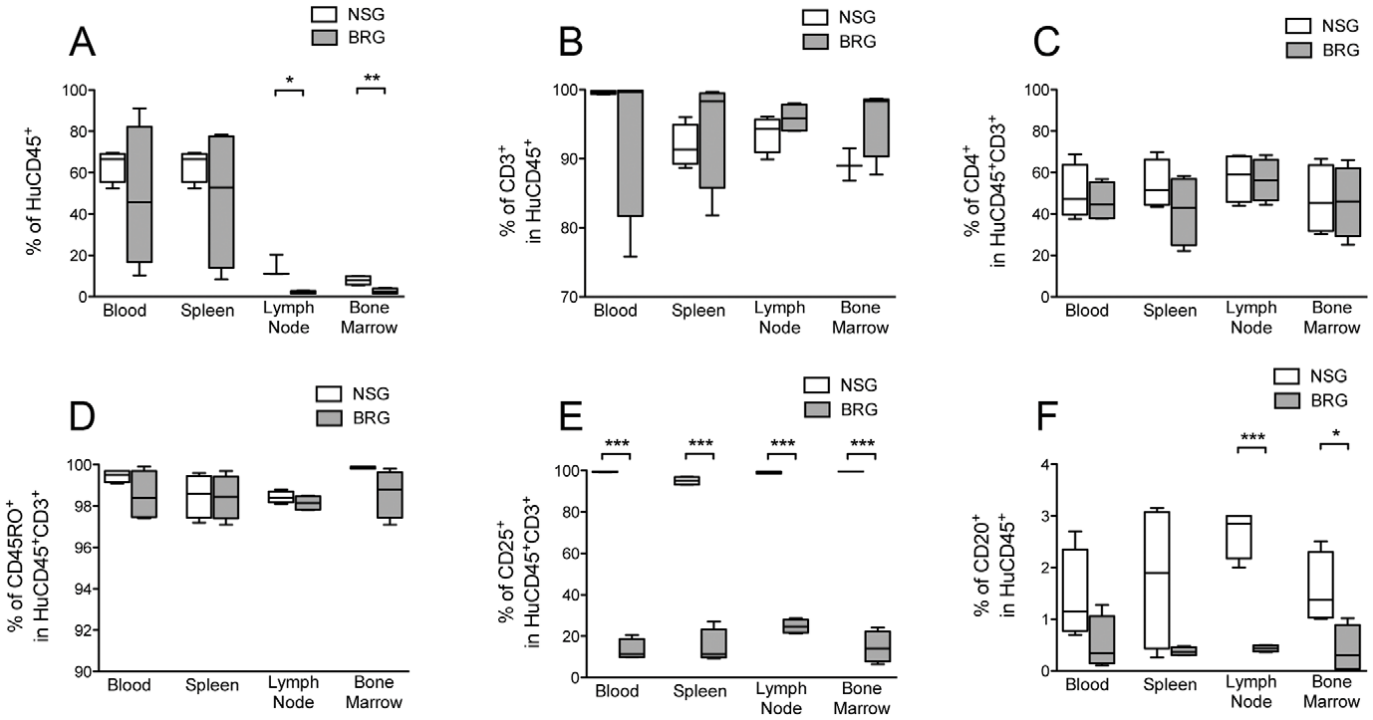
Human lymphocyte populations from Hu-PBMC NSG and BRG mice tissues harvested at xenogeneic Graft-versus-Host-Disease (GvHD). Peripheral blood, spleen, lymph nodes, and bone marrow were harvested at GvHD development in Hu-PBMC NSG and BRG mice and stained with anti-human specific antibodies for human (A) CD45, (B) CD3, (C) CD4, (D) CD25, (E) CD45RO, and (F) CD20 and quantified by flow cytometry. Tukey box-and-whisker graphs are shown. Unpaired t test was performed. Data are compiled from 3 independent experiments (*n* = 4 NSG and BRG mice respectively, 2 PBMC donors).

Previous studies have shown that the majority of engrafted human cells in immunodeficient mice (including NSG mice) develop an activated/memory CD45RO^+^ phenotype [18], [20]. We decided to monitor CD45RO, and an additional activation marker CD25, in the peripheral blood (Fig. 2D–E) and in the tissues (Fig. 4D–E) of Hu-PBMC NSG and BRG mice. CD45RO expression was consistent between the two strains, however, expression of CD25 on donor T-cells was significantly different in the blood at 5 weeks and in all tissues upon harvest, with >90% of T-cells in NSG mice expressing CD25 (Fig. 2D and 4E), this may reflect host-dependent differences in the degree or kinetics of T-cell activation. T-cell expression of CD69 was also measured and found to be similar at all time points (data not shown).

Upon harvest no differences were detected in the proportion of CD3^+^ T-cells in lymphoid tissues (Fig. 4B). One notable disparity between the two models was a significantly higher proportion of CD20^+^ B-cells in the lymph nodes (P<0.001) and bone marrow (P<0.01) of NSG mice (Fig. 4F).

Overall, these data show that NSG mice develop GvHD significantly faster than BRG mice, an important factor considering the use of these models as rapid tools for the assessment of novel therapeutics. We therefore selected the Hu-PBMC NSG model for all subsequent characterization.

### Tissues from Hu-PBMC NSG mice display a T-effector memory (T_EM_) phenotype

**Figure 5:**
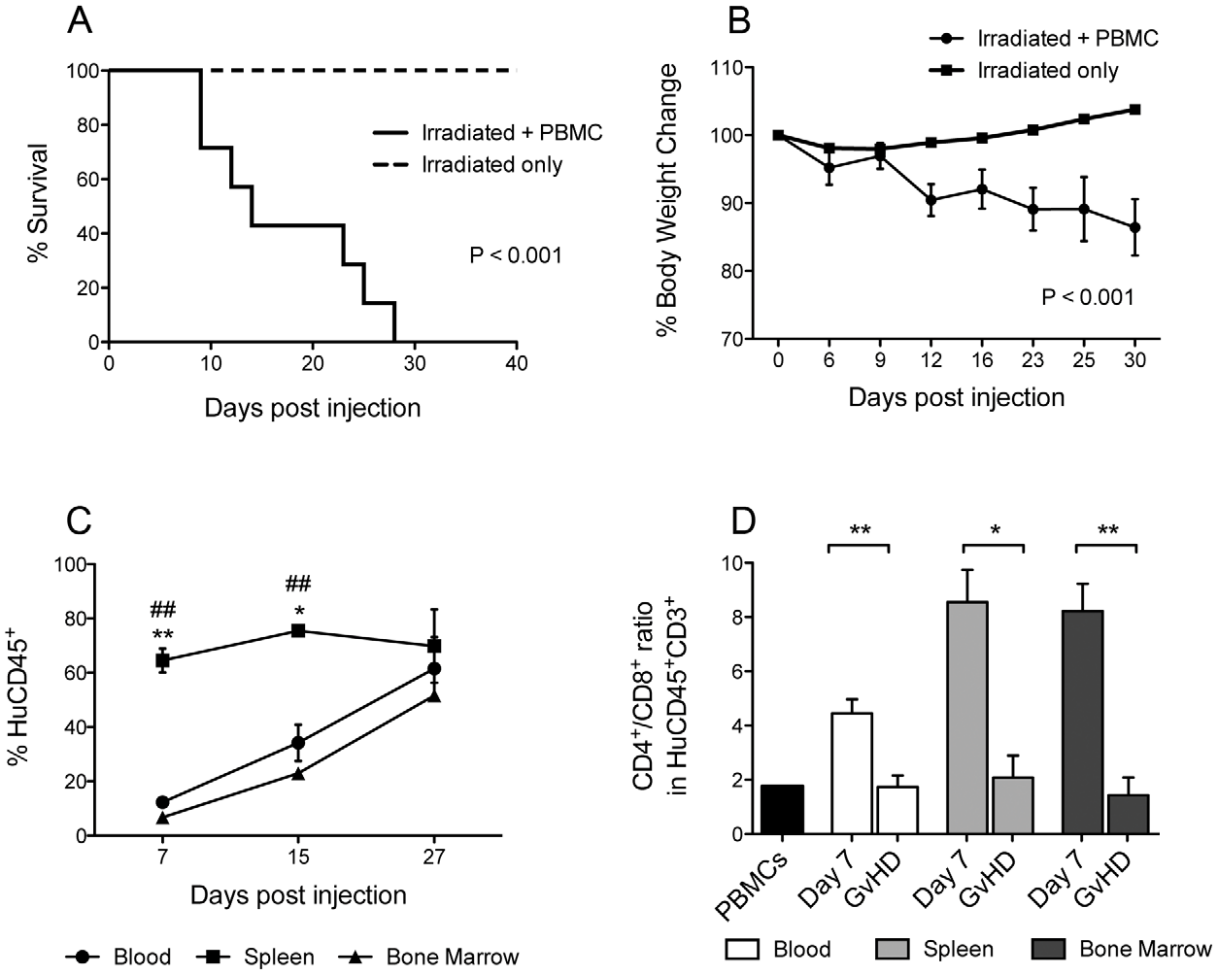
Sublethal irradiation accelerates xenogeneic Graft-versus-Host-Disease (GvHD) in Hu-PBMC NSG mice. 10^7^ freshly isolated PBMCs were injected intravenously via the tail vein into adult 6–12 week old NSG immunodeficient mice 24 hours post sub lethal irradiation (2.4 Gys). (A) Survival of irradiated Hu-PBMC NSG mice (MST = 14 days, *n* = 7, 2 PBMC donors). (B) The average weight is shown as a percentage of starting weight. Peripheral blood, spleen and bone marrow were harvested from irradiated Hu-PBMC NSG mice at the indicated time points and analysed for (C) human CD45 engraftment and (D) CD4:CD8 ratio. **Black bar represents human PBMC phenotype pre-injection.** Data represent the mean ±SEM and are compiled from 2 independent experiments (n = 7). (A) Log-rank Mantel-Cox Test, (B, D) unpaired t test, and (C) 2-way ANOVA, spleen vs blood (*,**) and spleen vs bone marrow (##).

Although Hu-PBMC NSG mice show gradual weight loss and eventual GvHD over a 50 day period (Fig. 1A and B), we decided to accelerate disease development by introducing a protocol of light irradiation prior to adoptive transfer of human cells [11] (Fig. 5A–B; MST = 14 days vs. 40 days in unconditioned Hu-PBMC NSG mice). This allowed us to study an even more optimized GvHD model, where disease development is significantly more rapid. We firstly examined the kinetics of human cell engraftment in tissues of irradiated Hu-PBMC NSG mice. Human CD45^+^ engraftment levels were significantly higher in the spleen at day 7 (P<0.01 vs blood and bone marrow) and day 15 (P<0.05 vs blood and P<0.01 vs bone marrow) but was then equal at day 27 (Fig. 5C). Comparison of the CD4:CD8 compartment within this human CD45^+^ population between early (day 7) and late (GvHD) time points revealed a significantly reduced ratio in all tissues examined (Fig. 5D). We have observed previously in patients with GvHD that phenotypic characterization of naïve, memory, and effector T-cell subsets can reveal important insights into the immunological basis of disease [21]. To determine the presence and/or proportions of T-cell subsets in Hu-PBMC NSG mice is important to understand the type of immune response that is associated with xeno-GvHD pathology in these humanized models. CD45RO, CD27, and CD62L were combined with anti-CD4 and anti-CD8 antibodies to analyze within the CD4^+^ and CD8^+^ populations the effectors (CD45RO^+^CD27^−^), the naïve (CD45RO^−^CD27^+^) to memory (CD45RO^+^CD27^+^) ratio, and the T_CM_ (CD45RO^+^CD27^+^CD62L^+^) to T_EM_ (CD45RO^+^CD27^+^CD62L^−^) ratio. Representative examples of gating strategies used to define T-cell subsets in mouse tissues are shown in Fig. S1.

**Figure 6:**
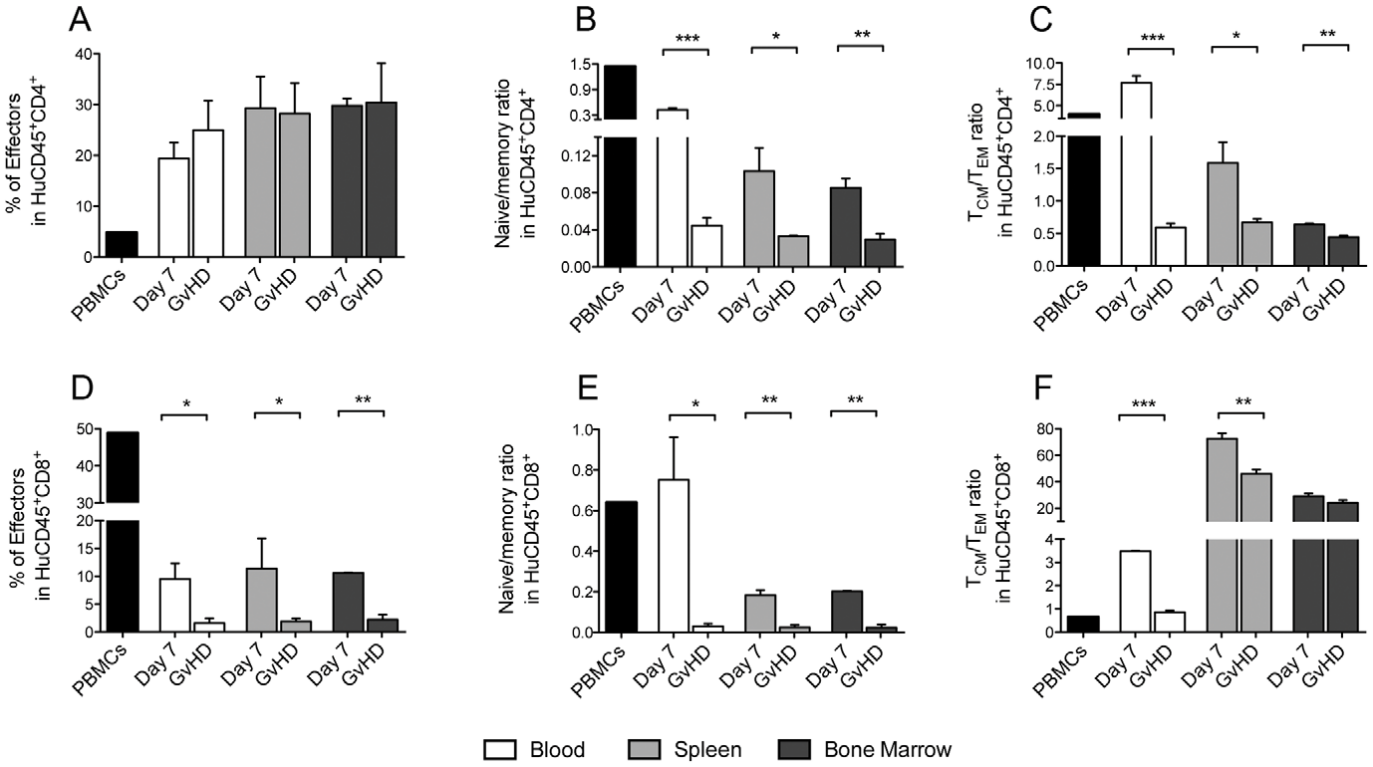
Engrafted T-cells in Hu-PBMC NSG mice display an effector memory tissue homing phenotype. Tissues were harvested from irradiated Hu-PBMC NSG mice at the indicated time points and T-cell subset compositions quantified: (A and D) CD45RO^+^CD27^−^ effectors, (B and E) naïve (CD45RO^−^CD27^+^) to memory (CD45RO^+^CD27^+^) ratio, and (C and F) central memory (CD45RO^+^CD27^+^CD62L^+^) to effector memory (CD45RO^+^CD27^+^CD62L^−^) ratio. **Black bars represent human PBMC phenotype pre-injection.** Unpaired t test was performed. Each data point represents the mean ±SEM and are compiled from 2 independent experiments (*n* = 7).

While there were no differences in the proportion of effector CD4^+^ T-cells present in the tissues (Fig. 6A), this was significantly reduced in the CD8^+^ T-cell pool at harvest after GvHD development (Fig. 6D). However, for both lineages the naïve:memory ratio was significantly lower at harvest as compared to day 7 (Fig. 6B and E). To further investigate this increased percentage of memory T-cells, we subdivided these into T_CM_ and T_EM_ to compare relative differences. Phenotypic analysis showed a markedly reduced T_CM_:T_EM_ ratio within the CD4^+^ fraction in all tissues analysed (Fig. 6C). This was also true for the CD8^+^ fraction in the peripheral blood and spleen (Fig. 6F). The increasing proportion of T-cells with an effector memory phenotype in Hu-PBMC NSG mice at GvHD development potentially suggests xenoantigen-driven survival and expansion. Interestingly, the ratio of T_CM_:T_EM_ cells remains high in the lymphoid organs suggesting that central memory cells in these organs may act as a reservoir for the expansion of effector memory cells in the blood.

### Engrafted T-cells express cutaneous lymphocyte antigen (CLA) and home to murine skin

**Figure 7:**
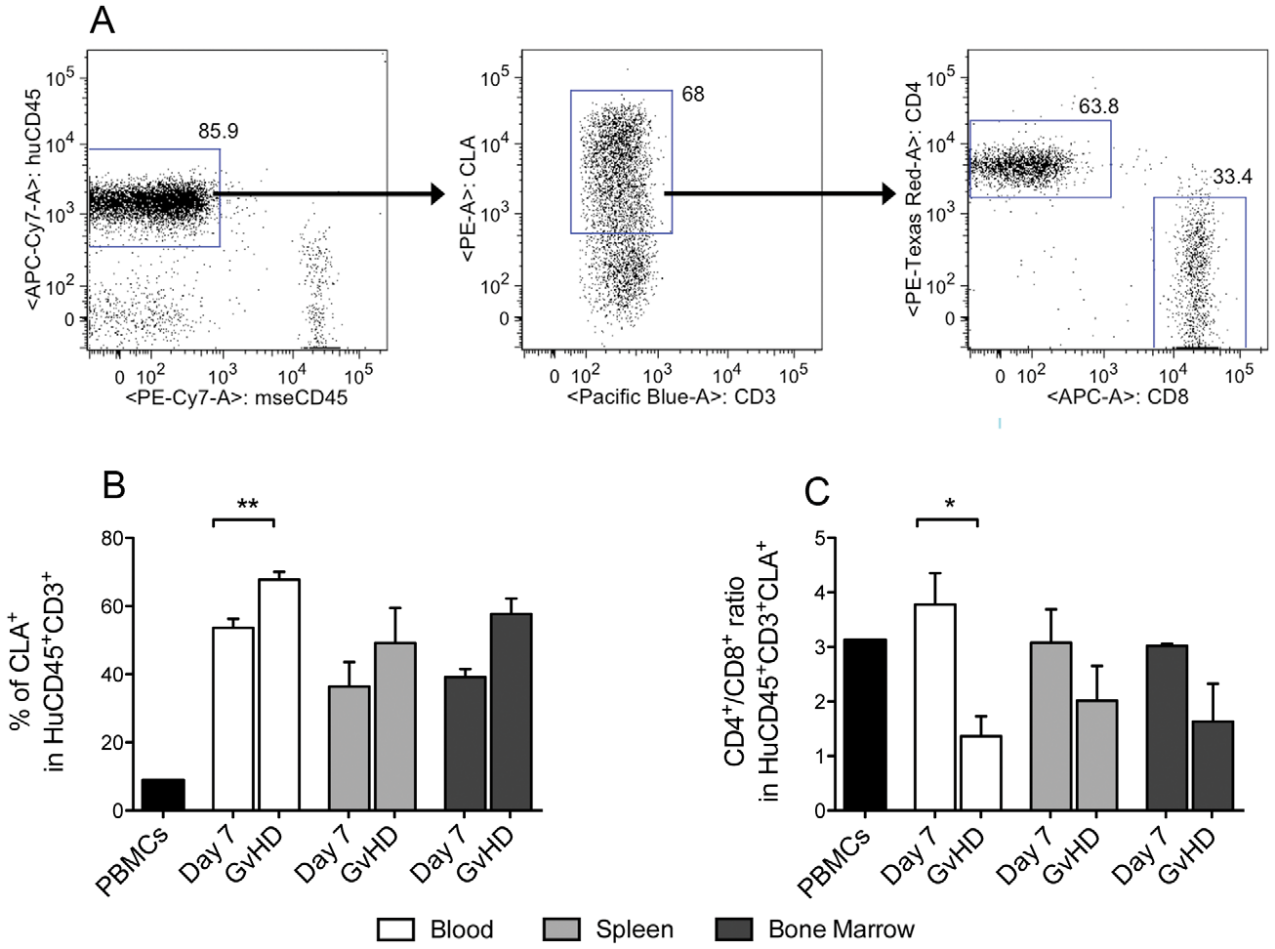
Human T-cells in Hu-PBMC NSG mice display skin homing capabilities. Peripheral blood, spleen and bone marrow were harvested from irradiated Hu-PBMC NSG mice at the indicated time points. Representative gating of peripheral blood is shown in (A). CD3^+^ T-cells were analysed for (B) CLA expression and (C) CD4 to CD8 ratio of CLA expressing CD3^+^ T-cells. **Black bars represent human PBMC phenotype pre-injection.** Unpaired t test was performed. Each data point represents the mean ±SEM and are compiled from 2 independent experiments (*n* = 7).

In human-into-mouse xenogeneic GvHD there have been reports of human immune cell infiltration into peripheral tissues such as the skin [6], [11], [22]–[24], mimicking the human form of the disease [25]. In some mice, we also observed signs of cutaneous GvHD pathology that manifested mainly as hair loss (data not shown). We therefore hypothesized that T-cell expression of CLA, which mediates specific skin homing of circulating peripheral blood T-cells [26]–[29], may increase during the course of GvHD in Hu-PBMC NSG mice. Indeed, in the peripheral circulation (but not in the spleens or bone marrow) there was significantly higher CLA expression among CD3^+^ T-cells at the point of GvHD development (P<0.01; Fig. 7B). To further characterise this expression, we analysed the composition of CLA expressing CD3^+^ T-cells and observed a decreased CD4:CD8 ratio, that again only reached statistical significance in the peripheral circulation (P<0.05; Fig. 7C). In addition, the skin homing chemokine receptors CCR4 and CCR6 [30] were also expressed on T-cells of Hu-PBMC NSG mice (Fig. S2A and B), further supporting our hypothesis. We then performed immunohistochemical staining of CD45^+^ cells in murine skin (Fig. S2C) showing that these engrafted human cells can infiltrate into the dermis (also confirming previous reports showing mouse skin infiltration in this model [11]).

## Discussion

Immunodeficient mice with the targeted IL-2Rγ^null^ mutation, namely the NSG and BRG, are rapidly becoming the superior choice of host for the creation of “Human Immune System” mice via reconstitution with human hematopoietic stem cells [4], [5]. These strains also support high levels of human PBMC engraftment without the requirement of pre-conditioning regimens such as total body irradiation or depletion of host macrophages, that were pre-requisites for successful engraftment in previous generations of immunodeficient mice [6], [11], [17]–[19]. In this study, we have performed the first comparative phenotypic characterization of human PBMC engraftment and kinetics in the two most commonly used mouse models of xenogeneic GvHD, Hu-PBMC NSG and BRG mice.

Human PBMCs from the same donors induced GvHD in both strains consistently, however at different rates. NSG mice are superior models in this respect as they develop GvHD significantly faster than BRG mice. Investigating the kinetics of human cell engraftment revealed that while human CD45^+^ levels increased in the peripheral circulation of both strains, this was significantly higher in NSG hosts. Furthermore, the expansion of the human compartment was also significantly faster in NSG mice as levels of human CD45 were >40% at five weeks post transfer of PBMC, at which point BRG mice only harboured <10%. It has previously been suggested that the development of xenogeneic GvHD in lymhodepleted hosts is driven by recognition of host MHC and is therefore dependant upon relatively high levels of T-cell engraftment [31]. Evaluation of T-cell kinetics indicated relatively lower proportions and of CD3^+^ cells in the periphery and spleens of BRG mice early time points post adoptive transfer of human cells. These data potentially suggest that the delayed time for GvHD development in the Hu-PBMC BRG model may be attributed to differences in the kinetics of human cell expansion and overall differential proportion of CD3^+^ T-cell engraftment.

T-cells of the CD4 and/or CD8 lineage are principle effectors of the immune response [2], [3], however the heterogeneity of the T-cell pool is scarcely taken into account in human GvHD studies. T-cells may be broadly divided into those that are antigen-inexperienced (naive T-cells) and antigen-experienced (which include effector and memory T-cells) [32], [33]. Memory T-cells can be further subdivided into effector memory T-cells (T_EM_) that preferentially migrate to inflamed peripheral tissues and are the major effector arm. Most cases of human acute GvHD begin with cutaneous lesions that often appear 2–3 weeks post HSCT, which result from the immune reactivity and homing of transplanted cells to target organs [25]. Conversely, central memory T-cells (T_CM_) efficiently home to secondary lymphoid organs and have little to no effector activity [34]. We have recently performed a longitudinal analysis of lymphocyte subset reconstitution in the peripheral blood of patients undergoing HSCT, showing that an imbalance of effector and regulatory T-cells is an indicative signature of GvHD in the context of T-cell depletion with campath 1H and cyclosporine prophylaxis [21]. Furthermore, in patients that develop acute GvHD, higher numbers of peripheral effector and effector memory CD4^+^ T-cells were prevalent. This ‘signature’ of GvHD is specific to a particular HSCT treatment regimen, as other treatments have been shown to lead to distinct pathways of pathology (as shown in [2] and [3] where CD8^+^ T-cells are essential). It seems that virtually any T-cell subset can potentially contribute to GVHD since the type of immune response that dominated probably depends on ‘environment’. Zheng et al.,[35] have shown that CD8^+^ T_CM_ cells are capable of inducing GvHD whereas other studies have shown a relative inability of memory T-cells to cause GvHD [36]. Indeed, in our lightly irradiated Hu-PBMC NSG model, we observed an increase of engrafted human cells with a T_EM_ tissue homing phenotype, both within the CD4 and CD8 lineages. Despite a reduction in the CD8^+^ T_CM_ to T_EM_ ratio we observed large proportions of these cells still present upon harvest (∼40∶1), suggesting that CD8^+^ T_CM_ cells may also be involved in xeno-GvHD response in this model. Concomitant with an enlarged presence of T_EM_ cells was an increased proportion of CLA^+^ T-cells present in the peripheral circulation, that were capable of homing to murine skin. A major aim of our study was to identify an optimal strain for the study of xenogeneic GvHD, and we selected the NSG mice to further characterize as disease onset was more rapid in this strain and displayed increased levels of human cell expansion as compared to BRG mice. Although we focused on characterizing T-cell subsets in irradiated Hu-PBMC NSG mice, it would also be interesting and informative to study the effect of irradiation pre-conditioning in BRG mice. Given the rapid development of disease and higher T-cell engraftment, NSG mice would be the strain of choice for most researchers studying T-cell driven xeno-GvHD in human PBMC engrafted mice.

In conclusion, our study has characterized T-cell subsets involved in the xeno-immune response of Hu-PBMC NSG and BRG mice. We decided to focus on defining the nature of the T-cell response associated with xeno-GvHD pathology in these humanized mice and show that Hu-PBMC NSG mice display a prevalence of tissue homing T_EM_ cells. Overall, illustrating that there are sufficient similarities between these models and what is known to occur in the clinical HSCT setting. These small animal models that can mimic certain aspects of clinical GvHD will therefore be useful for evaluating control of human T_EM_-cell driven GvHD by novel interventional therapies.

## Supporting Information

Figure S1 **Representative gating strategies to identify memory T-cells in human PBMCs and Hu-PBMC NSG mice.** Within the human CD45^+^ mouse CD45^−^ human CD3^+^ gate, CD4^+^ and CD8^+^ cells (top panel in A and B) were then selected to analyze CD4^+^ T-cell subsets (middle panel in blue box) and CD8^+^ T-cell subsets (bottom panel in red box). Subset classifications were as follows; effectors: CD45RO^+^CD27^−^; naïve: CD45RO^−^CD27^+^; and memory: CD45RO^+^CD27^+^. The memory population was then further subdivided into central memory: CD45RO^+^CD27^+^CD62L^+^ and effector memory: CD45RO^+^CD27^+^CD62L^−^.
(TIF)

Figure S2 **Human T-cells in Hu-PBMC NSG mice express skin homing chemokine receptors.** Peripheral blood, spleen and bone marrow were harvested from irradiated Hu-PBMC NSG mice at the indicated time points and CD3^+^ T-cells were analysed for (A) CCR4 and (B) CCR6 expression among engrafted CD3^+^ T-cells. (C) Human CD45^+^ cells were detected via immunohistochemical staining of mouse skin from irradiated Hu-PBMC NSG mice. **Black bars represent human PBMC phenotype pre-injection.** Unpaired t test was performed for each tissue that showed no statistically significant differences. Each data point represents the mean ±SEM and are compiled from 2 independent experiments (*n* = 7).
(TIF)
