# Supplementary figures and images for: Xenogeneic Graft-versus-Host-Disease in NOD-scid IL-2Rγnull Mice Display a T-Effector Memory Phenotype
Source: PLoS One. 2012 Aug 28;7(8):e44219. doi: 10.1371/journal.pone.0044219 (PMC3429415; doi:10.1371/journal.pone.0044219)

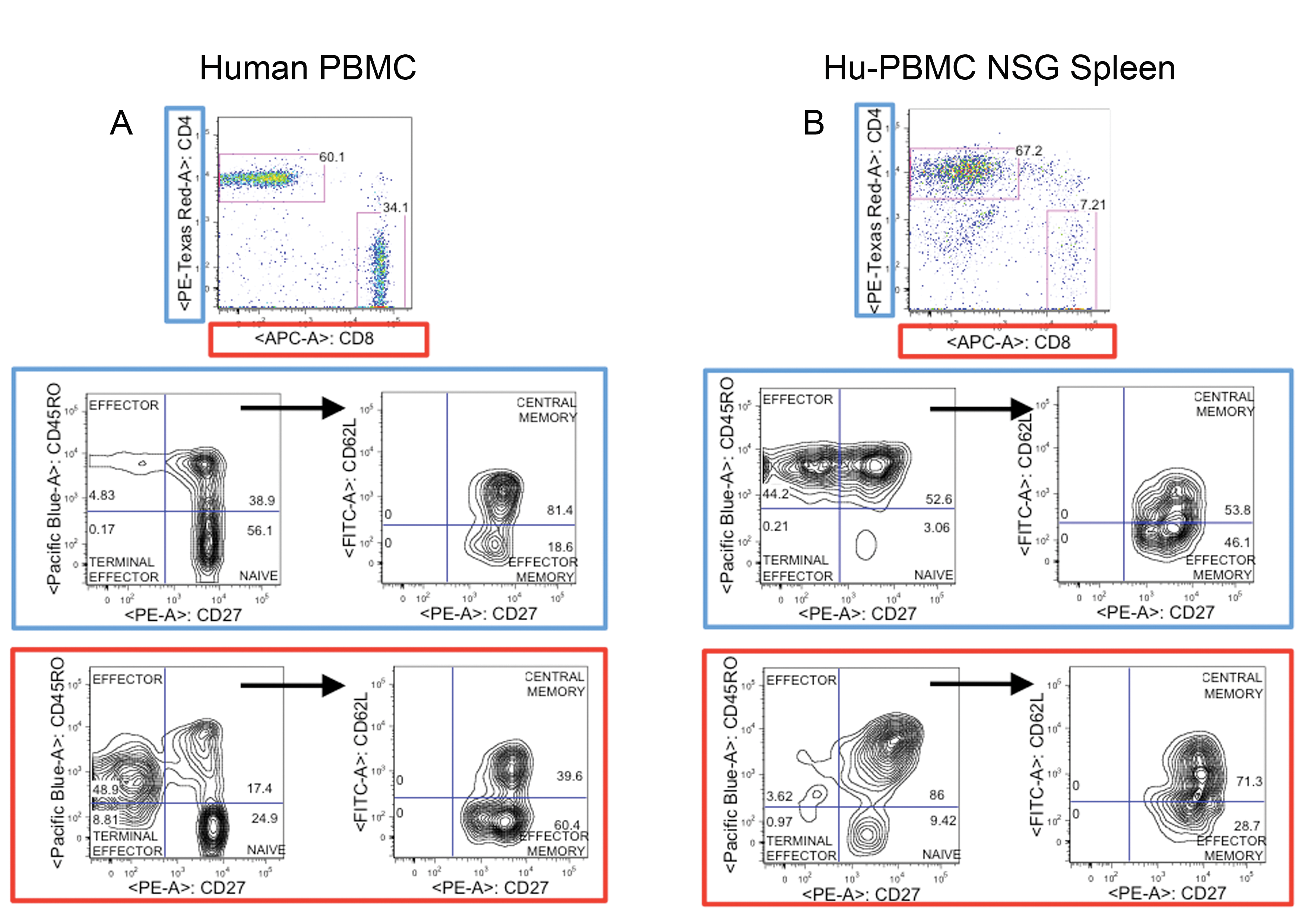

Supplement: Figure S1 — Representative gating strategies to identify memory T-cells in human PBMCs and Hu-PBMC NSG mice. Within the human CD45+ mouse CD45− human CD3+ gate, CD4+ and CD8+ cells (top panel in A and B) were then selected to analyze CD4+ T-cell subsets (middle panel in blue box) and CD8+ T-cell subsets (bottom panel in red box). Subset classifications were as follows; effectors: CD45RO+CD27−; naïve: CD45RO−CD27+; and memory: CD45RO+CD27+. The memory population was then further subdivided into central memory: CD45RO+CD27+CD62L+ and effector memory: CD45RO+CD27+CD62L−. (TIF) [file pone.0044219.s001.tif]

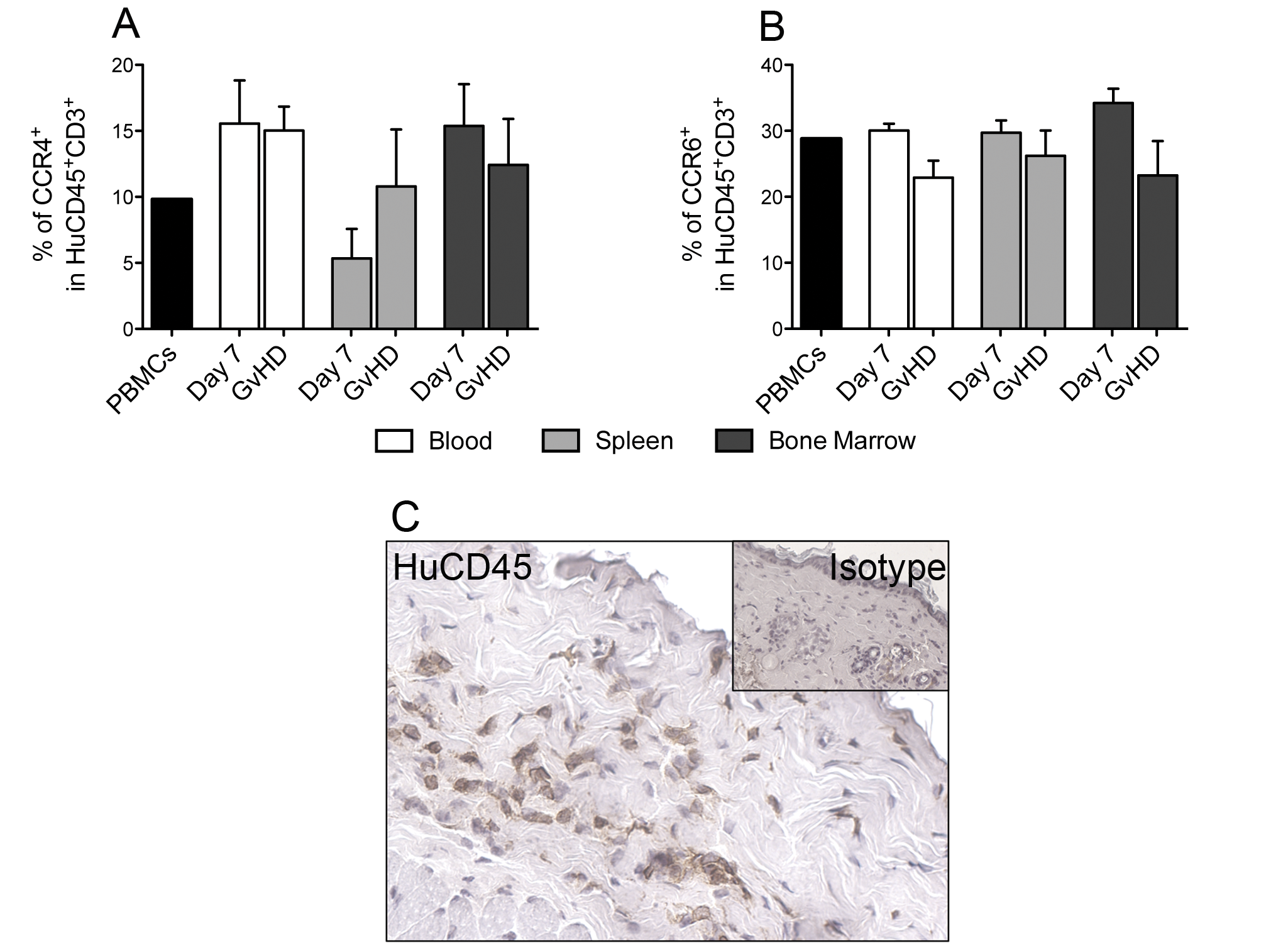

Supplement: Figure S2 — Human T-cells in Hu-PBMC NSG mice express skin homing chemokine receptors. Peripheral blood, spleen and bone marrow were harvested from irradiated Hu-PBMC NSG mice at the indicated time points and CD3+ T-cells were analysed for (A) CCR4 and (B) CCR6 expression among engrafted CD3+ T-cells. (C) Human CD45+ cells were detected via immunohistochemical staining of mouse skin from irradiated Hu-PBMC NSG mice. Black bars represent human PBMC phenotype pre-injection. Unpaired t test was performed for each tissue that showed no statistically significant differences. Each data point represents the mean ±SEM and are compiled from 2 independent experiments (n = 7). (TIF) [file pone.0044219.s002.tif]
